# ‘If one doesn't happen, the other will’: forensic mental health service patients’ experiences of co-occurring self-harm and aggression

**DOI:** 10.1192/bjo.2024.834

**Published:** 2025-01-22

**Authors:** Matina Shafti, Peter Taylor, Andrew Forrester, Louise Robinson, Sandeep Mathews, Daniel Pratt

**Affiliations:** Division of Psychology and Mental Health, School of Health Sciences, Manchester Academic Health Science Centre, University of Manchester, Manchester, UK; and School of Human Sciences and Institute for Lifecourse Development, University of Greenwich, London, UK; Division of Psychology and Mental Health, School of Health Sciences, Manchester Academic Health Science Centre, University of Manchester, Manchester, UK; Division of Psychological Medicine and Clinical Neurosciences, School of Medicine, Cardiff University, Cardiff, UK; Division of Psychology and Mental Health, School of Health Sciences, Manchester Academic Health Science Centre, University of Manchester, Manchester, UK; and Lancashire and South Cumbria NHS Foundation Trust, Preston, UK; Greater Manchester Mental Health NHS Foundation Trust, Manchester, UK

**Keywords:** Self-harm, aggression, co-occurrence, suicide, forensic mental health

## Abstract

**Background:**

Co-occurring self-harm and aggression (dual harm) is particularly prevalent among forensic mental health service (FMHS) patients. There is limited understanding of why this population engages in dual harm.

**Aims:**

This work aims to explore FMHS patients’ experiences of dual harm and how they make sense of this behaviour, with a focus on the role of emotions.

**Method:**

Participants were identified from their participation in a previous study. Sixteen FMHS patients with a lifetime history of dual harm were recruited from two hospitals. Individuals participated in one-to-one, semi-structured interviews where they reflected on past and/or current self-harm and aggression. Interview transcripts were analysed using reflexive thematic analysis.

**Results:**

Six themes were generated: self-harm and aggression as emotional regulation strategies, the consequences of witnessing harmful behaviours, relationships with others and the self, trapped within the criminal justice system, the convergence and divergence of self-harm and aggression, and moving forward as an FMHS patient. Themes highlighted shared risk factors of dual harm across participants, including emotional dysregulation, perceived lack of social support and witnessing harmful behaviours. Participants underlined the duality of their self-harm and aggression, primarily utilising both to regulate negative emotions. These behaviours also fulfilled distinct purposes at times (e.g. self-harm as punishment, aggression as defence). The impact of contextual factors within FMHSs, including restrictive practices and institutionalisation, were emphasised.

**Conclusions:**

Findings provide recommendations that can help address dual harm within forensic settings, including (a) transdiagnostic, individualised approaches that consider the duality of self-harm and aggression; and (b) cultural and organisational focus on recovery-centred practice.

Forensic mental health services (FMHSs) specialise in the assessment, treatment, management and rehabilitation of mentally unwell individuals who are mostly within the criminal justice system (CJS). Self-harm and aggression present a particular concern within these settings, with up to 88 and 60% of FMHS patients engaging in these behaviours, respectively.^1,2^ Although some engage in self-harm or aggression (i.e. sole harm), a group of individuals engage in both, referred to as dual harm.^3^ Compared with other populations (e.g. community-dwelling groups, prisoners), the highest prevalence of dual harm has been reported among FMHS patients, with up to 56% engaging in this behaviour.^4^ To the best of our knowledge, there are currently no evidence-based guidelines for how to manage dual harm, with current guidance limited to sole harm.^5,6^ This is concerning, given that those who dual harm have been found to be more likely to experience negative outcomes than those who engage in sole harm, including higher risk of unnatural death (e.g. fatal unintentional self-poisoning), spending 40% longer in prison and being more likely to experience restrictive practices (e.g. punishment, segregation).^3,7,8^ Additionally, these individuals are significantly more likely to experience various environmental, psychological and sociodemographic risk factors, including difficulties in self-regulation, low self-control and early adverse events.^9,10^ Individuals who dual harm further show riskier behavioural patterns, including more severe self-harm, being less likely to stop harmful behaviours and higher rates of in-prison incidents.^7,11,12^ Growing evidence has highlighted the association between aggression and self-harm, including suicidal behaviour, highlighting the importance of exploring the link between these behaviours in greater depth.^13^

To the best of our knowledge, no previous study has qualitatively explored FMHS patients’ experiences of dual harm. Pickering and colleagues^14^ investigated dual harm among six male prisoners. Findings identified various factors linked to dual harm, including early adverse events, challenging environments, difficulties with identity and distressing psychological states. The specific behaviour the individual engaged in was influenced by circumstance, opportunity and importance placed on physical pain in providing escape from internal states. Hemming and colleagues^15^ investigated alexithymia and harmful behaviours among 15 male prisoners. Participants reported that a build-up of the same emotions led to both self-harm and aggression. The specific harmful behaviour the individual engaged in was linked to their current and historical circumstances. Such findings highlight the role of emotions and contextual factors in co-occurring self-harm and aggression, as further outlined by Shafti and colleagues’^16^ cognitive emotional model of dual harm.^14,15^ In light of the distinct context of FMHSs and the unique dual stigma faced by patients within these settings – having a mental disorder and being within the CJS – experiences of dual harm may differ between prisoners and FMHS patients. Therefore, the present study aimed to qualitatively explore FMHS patients’ experiences of dual harm and how they make sense of this behaviour.

## Method

### Participants

The authors assert that all procedures contributing to this work comply with the ethical standards of the relevant national and institutional committees on human experimentation and with the Helsinki Declaration of 1975, as revised in 2008. All procedures involving human patients were approved by Wales Research Ethics Committee 6 (approval number 21/WA/0168).

Participants’ eligibility was based on their participation in a prior study examining the role of psychopathy and emotional dysregulation in dual harm. Individuals were eligible if they were an FMHS patient, aged 18 years or older and had a lifetime history of self-harm and aggressive behaviour. There were no temporal restrictions placed on when these behaviours had to begin or occur. Self-harm was defined as acts of self-poisoning or self-injury, regardless of suicidal intent,^17^ whereas aggression was defined as intentional physical harm toward others.^18^ Exclusion criteria included lacking sufficient English language skills and mental capacity to provide informed consent, being a patient within the brain injury service or deemed by staff as posing too great a risk to themselves or others to participate safely. Written consent was obtained from all participants.

Sixteen participants from two FMHSs were recruited (Table 1). Participants were from low (*n* = 6) and medium secure (*n* = 10) wards. Fourteen identified as male and two as female. Ages ranged from 24 to 58 years (mean 40, s.d. = 10.92). Information regarding ethnicity and age were self-reported by participants, and information about offence and diagnosis were obtained from hospital clinical records.

**Table 1:** Demographic characteristics of participants

Demographic characteristics	*n*
Ethnicity	
White British	13
Black British	1
White and Black African	1
Persian	1
Diagnoses^a^	
Schizophrenia	9
Schizoaffective disorder	3
Personality disorder	3
Substance use disorder	3
Psychotic depression	1
Index offence^a^	
Violent^b^	12
Arson	4
Burglary and theft	1
Robbery and battery	1

a. Some participants had more than one diagnosis and offence, therefore totals reflect a higher number than the sample size.

b. Violent offences include murder, attempted murder, manslaughter, assault and sexual violence.

### Data collection

Participants took part in one-to-one, semi-structured interviews in a private room within their ward, based on a topic guide with open-ended questions (Supplementary Material 1 available at https://doi.org/10.1192/bjo.2024.834). Interviews explored experiences of emotions, self-harm, aggression and upbringing. As done in previous research, during the interview, participants were provided the opportunity to draw the emotions they experienced before engaging in harmful behaviours.^15^ All interviews were audio recorded and transcribed (17–56 min, mean duration 31 min). Following Braun and Clarke's^19^ recommendation, data quality was constantly reviewed during data collection to determine sample size. Interviews were conducted by M.S., a psychology PhD student with experience of researching harmful behaviours and qualitative methods.^20^

### Data analysis

Reflexive thematic analysis was conducted to generate themes.^21^ Sixteen interviews were considered sufficient, as they told a ‘rich, complex and multi-faceted story about patternings’, which allowed the research question to be addressed.^19^ A combination of inductive and deductive analysis was utilised to explore participants’ experiences (inductive), while also applying a theoretical lens based on existing theory and evidence (deductive). For example, because of the evidence base for emotional dysregulation as a contributing factor to harmful behaviours, as well as the link between self-harm and aggression, the topic guide explored participants’ experiences of emotions, as well as the potential link between self-harm and aggression.^13,22^ At the end of the interview, participants were given the opportunity to discuss any other things that they thought might be relevant. Semantic and latent analysis were applied to consider participants’ own accounts and the underlying meaning attached to these. An experiential framework was broadly adopted, focusing on participants’ experiences and how they understood their dual harm. Given evidence for the role of social constructs/contexts in dual harm, this was underpinned by a critical realist approach, recognising the impact of these on experiences of reality.^14,15^

M.S. conducted the analysis according to Braun and Clarke's^21^ thematic analysis phases. First, transcripts were read multiple times, while making reflective notes. Coding was then performed and labels written beside relevant texts. Grouped codes formed initial themes, representing central concepts capturing patterns of meaning. These were discussed with M.S.'s supervisory team (D.P., P.T. and A.F.) and transcripts were re-examined. Subsequently, themes were finalised, labelled and defined. Finally, the analysis was written up. During write-up, it became evident that certain themes shared greater patterns of meaning than initially thought, resulting in their grouping under one theme.

## Results

**Fig. 1:**
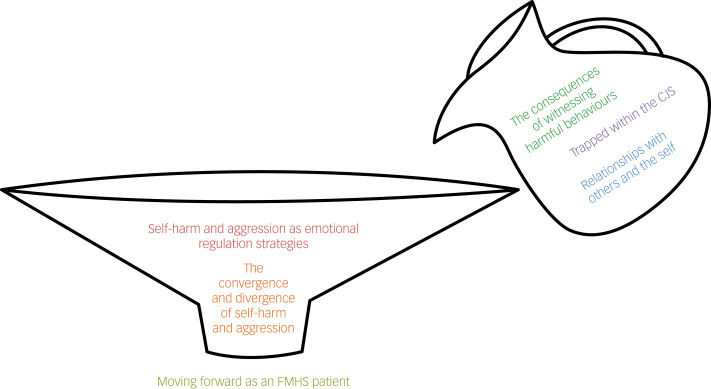
Thematic map. Themes related to internal and external adversities faced by patients, such as witnessing harmful behaviours, self-worthlessness and feelings of institutionalisation, are contained within the jug. These adversities interact and load onto each other, causing an overflow into the funnel, representing the compounding psychological distress experienced by individuals. This accumulation of adversities leads to the use of self-harm and aggression, both as shared (e.g. for emotional regulation) and distinct strategies (e.g. self-punishment) to manage such distress. ‘Moving forward as an FMHS patient’ is positioned at the end of the funnel, to represent the patient's journey toward recovery, having largely overcame the challenges linked to the other themes. The narrowing of the funnel symbolises how patients filter out their maladaptive behaviours and adopt healthier coping mechanisms. CJS, criminal justice system; FMHS, forensic mental health service.

The following themes were generated (Fig. 1): theme 1, self-harm and aggression as emotional regulation strategies; theme 2, the consequences of witnessing harmful behaviours; theme 3, relationships with others and the self; theme 4, the convergence and divergence of self-harm and aggression; theme 5, trapped within the CJS and theme 6, moving forward as an FMHS patient.

The data have been edited to provide context and remove irrelevant content ([ … ]). Personal identifiable information was removed from quotes and participants were provided with pseudonyms.

### Theme 1: self-harm and aggression as emotional regulation strategies

This theme captures how participants largely expressed using self-harm and aggression as a way to regulate intense, turbulent negative emotions – typically sadness, anger and frustration. All patients acknowledged challenges in emotional regulation, including understanding emotions, employing adaptive coping strategies and controlling impulsive behaviours. Although negative feelings were at times managed with adaptive methods (e.g. isolating, listening to music), participants turned to dual harm when emotions accumulated and felt like ‘too much to handle’ (Garry). In the absence of helpful emotional regulation skills, self-harm and aggression were likened to medication or drugs, necessary to ‘treat’ strong emotions: ‘It's [self-harm] like having a tablet for it [frustration]’ (Leo). Harmful behaviours helped participants in various ways, including providing a ‘distraction’, ‘adrenaline rush’ (Oscar) and calmness: ‘Being violent was like a comedown, because you're up there and then when I was violent, it was like an anti-climax… I felt calm’ (Charlie).

Although harmful behaviours were perceived as initially effective, participants acknowledged that these were ultimately unhelpful and ‘wrong’ (Leo). Nevertheless, participants continued their dual harm, attributed to an inability to control harmful urges. The impulsive nature of their self-harm and aggression led to these behaviours as ‘automatic’ (Francesca) habitual responses to negative feelings, where patients became ‘dependant’ (Rebecca) on them to feel better. Nick described feeling like he ‘lost control of my body’ before engaging in dual harm, whereas Craig explained that the urge to dual harm ‘pushes you forward, like egging you on’. In this way, participants justified their ongoing harmful behaviours by attaching a lack of personal agency to them. Similarly, participants emphasised that their dual harm did not occur in a vacuum. Rather, a multitude of adverse events caused a ‘downward trajectory’ (Leo), in which it was deemed inevitable that dual harm would be used to manage the ‘snowball’ (Simon) and ‘build-up’ (Richard) of emotions.

Over time, participants felt less in control of their dual harm: ‘It would feel like I had to do it [self-harm] more than I wanted do, that I didn't have a choice’ (Rebecca). Harmful behaviours would ‘spiral’ (Robbie), becoming more severe and harder to control, attributed to the behaviour not fulfilling its goal of emotional regulation in its initial form: ‘Superficial scratches wasn't doing it for me. I wouldn't feel the pain, and then it became deeper and worse because I felt the deeper the cut, the bigger the relief’ (Oscar). Participants often perceived the impulsive nature of their dual harm as a symptom of their mental health, stating ‘it's part of my illness’ (Robbie).

### Theme 2: the consequences of witnessing harmful behaviours

This theme reflects how participants related witnessing self-harm and aggression, particularly among family, friends and subsequently within forensic settings, to their own experiences of harmful behaviours. Repeatedly observing these acts led to their normalisation as a way to deal with emotions. Participants perceived behavioural norms as contextual, highlighting that their risk of dual harm decreased in the community: *‘*When I'm in a prison environment, I can self-harm, I can assault, I can be a complete nobhead, but in the community I'm sound… It's like when you take me out of that normality and put me in an environment where it's restrictive and more violent, my mentality completely changes’ (Logan).

Participants minimised responsibility over their harmful behaviours and directed this toward the cultural dynamic within forensic settings. Dual harm was often perceived as acceptable given that their peers within prisons and FMHSs engaged in this behaviour. Repeatedly witnessing harmful behaviours further led to their desensitisation, leading to a lack of emotional or mental strain in response to these acts: ‘I've become so desensitised that I can watch like others do it [violence] … I'm just numb to it [ … ] seeing violence around me growing up's made me desensitised and more likely to do it’ (Logan). Simon describes that not witnessing aggression for a long time gave him ‘more empathy towards’ victims, suggesting the process of desensitisation is reversible. Moreover, participants observed others having positive experiences when engaging in self-harm and aggression, resulting in their positive reinforcement. Consequently, dual harm was not only perceived as acceptable, but also helpful in dealing with negative emotions: ‘They're saying it [self-harm] makes your feelings go away, you feel relieved after it, so I thought I'd try it’ (Craig).

### Theme 3: relationships with others and the self

This theme reflects how participants’ relationships with others and themselves was both a risk and a protective factor for their dual harm. Many participants expressed lacking healthy relationships, attributable to difficulties with social skills or being surrounded by the wrong people. Consequently, this led to the perceived absence of a support system and ‘no one to talk to’ (Simon). Linking back to theme 1, this made it challenging to deal with emotions, with harmful behaviours used to ‘signal [distress] to other people’ (Simon): ‘I had no one. Mainly every time I self-harmed, I think it was just me needing a big hug or someone to say it's okay’ (Logan). In this way, self-harm was used to communicate distress and seek validation, comfort or reassurance. Given that FMHS patients’ main source of interaction is with hospital staff, these relationships greatly affected participants. Many highlighted that positive relationships with staff provided space to form healthy social bonds and communicate emotions in a healthy way, thereby reducing harmful behaviours.

For some, social relationships served as an indicator of their self-worth, and was thereby tied to their self-esteem. Positive and supportive relationships validated the individual, making them feel worthy and therefore, protected against self-harm: ‘At least I got some friends here who look after me, otherwise I think I'd have to harm myself. Having friends makes you think you're not inferior’ (Garry). On the other hand, a lack of positive relationships was internalised and attributed to self-perceived worthlessness and inability to form healthy bonds. Consequently, these individuals self-harmed to ‘beat’ themselves up (Leo): ‘I self-harmed because I hated myself. I would look back at my life and say, “you just fail at everything. No one loves you”. If I was punishing myself, I was doing something right’ (Francesca). In this way, self-harm was used as self-punishment. Such self-inflicted harm was often accompanied by a sense of achievement as patients believed they deserved to suffer, further validating their negative self-image.

### Theme 4: the convergence and divergence of self-harm and aggression

**Fig. 2:**
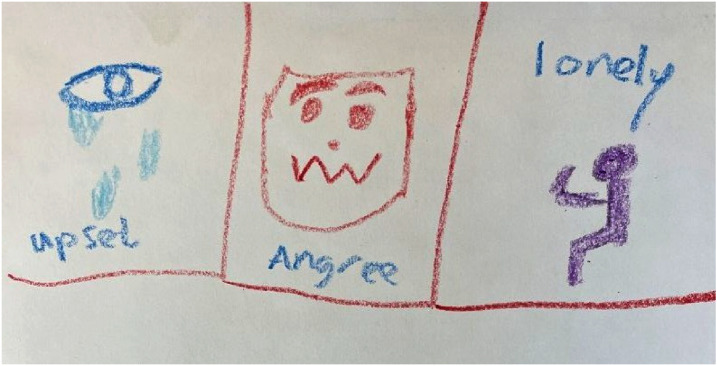
Oscar's drawing.

This theme reflects how most FMHS patients expressed a link between their self-harm and aggression, with these often preceded by the same emotions (e.g. sadness, anger, frustration) and circumstances: ‘The emotions I felt before [self-harm and aggression] came hand-in-hand [ … ] my self-harming and the violence happened all at the same time, from the same sort of circumstances’ (Simon). If it was not possible to engage in one of the behaviours (e.g. because staff would stop the individual from doing so), participants often expressed that they would inevitably engage in the other, implying a shared function between self-harm and aggression. Intersecting with theme 1, this function was often emotional regulation: ‘When I can't hurt myself, the emotions need to get out one way or another [ … ] If one doesn't happen [self-harm or aggression], the other will’ (Rebecca). Similarly, Oscar described how he would ‘flip’ his emotions ‘internally towards’ himself to avoid hurting others, indicating a common underlying drive. Oscar's drawing (Fig. 2) illustrates the shared internal states he experienced before his self-harm and aggression, including upset, anger and loneliness. As well as compensating for a lack of emotional regulation skills, self-harm and aggression further compensated for the lack of control participants felt over their lives, offering a semblance of power over themselves and others: ‘I felt a bit more in control [when self-harming] … I couldn't control what was going on around me, but I could control what I did to myself’ (Rebecca).

One of the harmful behaviours was typically predominant in the individual's life and began earlier. This behaviour was favoured because of its perceived usefulness and fewer consequences. Although some patients engaged in aggression to avoid hurting and scarring themselves, others used self-harm to avoid hurting another individual and being punished. Such consideration of how useful the behaviour would be in achieving their goals (i.e. behavioural utility) implies that patients made an active choice between self-harm and aggression. This suggests that these acts may not be as impulsive as initially suggested: ‘Both behaviours were a sense of me regaining control of the situation, but I'd self-harm more because it was more helpful. Less consequences. Less hurt and upset’ (Nick). The individual's ability to engage in a specific behaviour (i.e. behavioural accessibility) also influenced patients’ behaviours. Many expressed that they were unable to self-harm because of practical limitations (e.g. lacking an object to cause injury, staff stopping the behaviour), whereas seclusion meant they would have no one to direct aggression toward. Engaging in one of the behaviours decreased the risk of the other, whereas being unable to engage in one increased the risk of the other: ‘I kind of hurt people because they're stopping me hurting me [ … ] when I was in the community, there was nobody to stop me [self-harming] so there wasn't any aggression’ (Rebecca).

Although self-harm and aggression were largely expressed as interchangeable, they also served distinct functions. As highlighted in theme 3, self-harm was also used as self-punishment by those who struggled with self-worth: ‘You automatically do it if you feel that you need to punish yourself [ … ] I felt guilty if I didn't’ (Francesca). On the other hand, aggression was often justified as a response to provocation, particularly within forensic settings where it was necessary for ‘survival’ (Robbie): ‘I do act aggressive and I shouldn't be doing it, but I don't want to be made to look small [ … ] it's a dog eat dog world in prison. You have to do what you have to do to get by’ (Logan).

### Theme 5: trapped within the CJS

This theme captures how participants felt trapped within the CJS, which increased the risk of their dual harm. Most participants had been in FMHSs and prison for several years (mean duration of 4 years in FMHSs). Patients felt stuck within this system, ‘like a hamster trapped in a cage’ (Daniel), finding it difficult to visualise ‘a way out’ (Simon), leading to frustration and hopelessness: ‘For a long time, I saw the NHS as an enemy because I couldn't leave the places I was kept in. I didn't understand why I was there’ (Robbie). Such feelings were exacerbated by limitations placed on freedom (e.g. seclusion, having to request basic necessities, unable to access community leave), for what was often perceived to be unjustifiable reasons. Negative staff–patient interactions added to feelings of being ‘backed up into a corner’ (Robbie) in a system that was against them: ‘Three people who were twice as big as me each came and dragged me up the corridor and I just got injected. Those sort of scenarios happen so many times… it just made me lash out quicker [ … ] it made me defensive’ (Robbie). Participants also felt trapped in their future, constructing their identity of an FMHS patient as the ‘bottom of the ladder’ (Nick) in society. Many felt they lacked prospects within the community because of their institutionalisation and the stigma they faced: ‘I'm going to struggle getting a job with my history. No one's going to help you once they find out you've been here. I couldn't see a way out of it, so I kind of gave up on myself and that led to violent behaviour’ (Terry).

Those who had been in prison before FMHSs reported an absence of targeted interventions for their mental health and harmful behaviours within prison, suggesting a lack of sufficient support within these settings. Participants expressed that they should have been admitted to FMHSs earlier to receive the help they required, causing them to feel like the ‘system had let me down’ (Nick): ‘In prison it's like there's not enough done to stop people from being violent to yourself or others [ … ]; I should have been sent to [hospital name redacted] sooner’ (Simon). Participants’ self-harm and aggression within prison often became more severe as a ‘cry for help’ (Nick) to receive the care they required from staff: ‘[Self-harm and aggression being ignored] made it worse and more liable to carry on because if no one's listening to you, you just keep doing it’ (William). Such neglect of their harmful behaviours was suggested to have a detrimental impact on patients’ well-being, leading to further self-harm and aggression. In a system perceived to be unresponsive to their needs, patients’ dual harm often continued as a form of care-seeking behaviour from prison staff.

### Theme 6: moving forward as an FMHS patient

This theme captures how all participants contrasted their past to their present, expressing that their dual harm had now decreased or stopped. All patients reported dealing with emotions in a more helpful way, tied to developing emotional regulation skills and negative schema regarding harmful behaviours, largely through psychotherapy and access to healthier coping mechanisms. Participants described how engaging in therapy equipped them with invaluable tools for managing their emotions effectively. For example, techniques such as mindfulness and mental imagery were instrumental in identifying and challenging negative thought patterns and dealing with negative emotions. Moreover, accessibility to more positive coping mechanisms owing to greater freedoms within the hospital further helped patients. Patient activities, such as community leave and communal projects with other patients, offered opportunities for social connection and meaningful engagement, thereby alleviating feelings of boredom, isolation, frustration and containment that had often led to harmful behaviours: ‘I don't need to harm myself anymore … you're not sitting there bored. You go shopping, you go to the village [ … ] The trips out and everything help and I don't get aggressive. But because of that frustration of being locked up, then I get aggressive’ (Larry).

Progress within the hospital, typically reflected by an increase in freedom and privileges, also offered participants more confidence, a sense of agency and positive outlook for the future. For example, transitioning to less restrictive environments provided individuals with increased independence and normality outside the microcosm of the hospital, thereby mitigating feelings of hopelessness and frustration associated with confinement and aiding in recovery: ‘When you went to low secure, you didn't have to rely on staff so much. I found it helped because it gave you some independence, hope… I found it a lot easier dealing with them [negative] emotions’ (Nick). Participants expressed a strong sense of optimism and determination to move forward with their lives. Patients were ‘positive where my life's heading’ (Nick) and wanted to ‘repair the damage’ (Garry) they had caused in the past. This renewed hopefulness and vision of a future outside of the hospital meant participants were less likely to engage in dual harm as they were committed to continuing their progression into the community. In this way, participants linked the reduction and desistance of their dual harm to a multifaceted process of psychological and personal growth, facilitated by therapy, access to positive coping mechanisms and shifts in environmental dynamics that fostered a sense of agency.

## Discussion

Despite the lack of systemic acknowledgment of the duality of self-harm and aggression, participants in this study predominantly perceived these behaviours as interrelated, with both being primarily used to alleviate emotional distress. Participants highlighted various factors that contributed to their dual harm, including desensitisation and normalisation of harmful behaviours, perceived lack of social support and feeling trapped in the CJS. Through engaging in therapy, healthier coping mechanisms and increased sense of agency, patients were able to overcome the challenges linked to their dual harm, leading to the cessation or reduction of this behaviour.

Gratz and Roemer's^23^ conceptualisation of emotional dysregulation aligns with the challenges experienced by participants, including difficulties in ‘awareness, understanding, and acceptance of emotions; ability to control impulsive behaviors and engage in goal-directed behaviors when experiencing negative emotions; and flexible use of situationally appropriate strategies to modulate the intensity and duration of emotional responses, rather than to eliminate emotions entirely’.^24^ Participants experienced difficulties with identifying and understanding their emotions, controlling impulsive harmful behaviours and utilising healthy coping strategies when emotionally distressed. The above findings have been replicated in prisoners, indicating that those who dual harm have challenges in regulating their affect and may use this behaviour to escape or manage emotions.^14,15^ The experiential avoidance theory suggests that harmful behaviours are used to temporary relieve emotional distress arising from a lack of emotional regulation skills.^25^ This relief reinforces these behaviours, resulting in self-harm and aggression as conditioned and impulsive responses to negative affect.^25^ The experiential avoidance theory may account for participants’ construction of dual harm as an addictive and impulsive behaviour that they cannot control. Participants recognised that their harmful behaviours were ultimately unhelpful. However, perceiving dual harm as an uncontrollable addiction may have minimised their sense of agency, hindering the inhibiting effect of cognitive dissonance. The addictive nature of self-harm and aggression has been shown in previous research.^25^

Participants’ assessment of behavioural utility and accessibility suggests deliberate decision-making before dual harm. Similar findings have been observed among prisoners who engage in both behaviours.^14,15^ The above evidence aligns with Shafti and colleagues’^16^ cognitive–emotional model, suggesting that contextual factors and expectancies influence the individual's preferred behavioural response. Consistent with the above model, participants experienced dual harm as serving other functions beyond emotional regulation, including communicating distress.^16^ As found in previous research, participants used self-harm and aggression to express their feelings when they felt unable to do so verbally because of a lack of perceived social support.^15^ Additionally, dual harm provided participants with a sense of control, consistent with research demonstrating that harmful behaviours allow individuals to regain power over adverse situations.^14,26^ Such findings underscore an irony: the very behaviours participants believe they have no control over ‘paradoxically, affords them some degree of empowerment over their life situations’.^26^

Although self-harm and aggression served shared functions, they also fulfilled distinct purposes. For example, aggression was often expressed as a defensive response to provocation within prison or FMHSs. Nevertheless, this may suggest that those who engage in dual harm primarily exhibit reactive, rather than proactive, aggression. Reactive aggression is impulsive and driven by emotions in response to real/perceived threat, whereas proactive aggression is unemotional, with the goal of achieving rewards. Previous research has found that self-harm primarily occurs alongside reactive instead of proactive aggression.^27,28^ Reactive aggression may be more likely to co-occur with self-harm, as both are underpinned by strong emotions and emotional dysregulation.^27,28^ Therefore, although self-harm and aggression may serve distinct functions, emotional dysregulation could be a shared underlying mechanism in the context of dual harm. Nevertheless, it should be noted that participants often viewed aggression as a necessary act of defence in the hostile environment of forensic settings. Therefore, rather than solely attribute it to emotional dysregulation, it is important to consider the context within which dual harm occurs.

As found in prisoners who dual harm, witnessing others engaging in harmful behaviours contributed to the construction of dual harm as an acceptable, normalised and helpful way to deal with distress.^15^ In support of the above, it has been revealed that observing parental violence and having a family member who has self-harmed is more prevalent in those who have dual harmed compared with sole harm.^29^ Moreover, research has found a higher rate of suicidal behaviours in prisoners who have witnessed their peers engaging in such behaviours compared with those who have not.^30^ Continuous exposure to self-harm and aggression among other patients/prisoners and staff within forensic settings may perpetuate a cycle of reinforced harmful behaviours. As outlined in established models of self-harm and aggression (e.g. interpersonal theory of suicide^31^; general aggression model^32^), repeated exposure to fear-provoking stimuli (e.g. self-harm and aggression) desensitises individuals to pain and violence, thereby increasing the risk of harmful behaviours. Desensitisation may account for why participants’ dual harm became more severe over time, as they developed tolerance toward harmful behaviours in their initial, less severe form.^16^

Participants emphasised that their experiences cannot be separated from their context. As with previous research of forensic populations, participants expressed overriding feelings of hopelessness and frustration within the CJS, contributing to harmful behaviours as a coping mechanism.^33,34^ Such feelings may be particularly prevalent among FMHS patients who have been described as being ‘doubly deviant’ because of their dual stigma, being more likely to experience social exclusion and deprivation.^35^ In line with present findings, studies have highlighted that restrictive practices (e.g. seclusion, lack of leave) and negative staff–patient interactions increase the risk of harmful behaviours among psychiatric patients.^36,38^ This may especially be a concern for those who dual harm. Research has found that FMHS patients who have engaged in suicidal behaviour and violence are perceived by staff as difficult to treat, and such perceptions could lead to poorer patient progress.^39,40^ Such negative staff–patient interactions and restrictive practices can impede the development of independence, hope and non-patient identity, thereby leading to institutionalisation and slow patient recovery.^41^ The necessary tools are already in place within FMHSs to address such institutionalisation (e.g. social participation, community leave, rehabilitative programmes).^42^ However, participants’ emphasis on the prevailing atmosphere of inertia and restrictive culture suggests that these are often not experienced.

To better support patients, reducing restrictive practice and institutionalisation should remain a primary focus of mental health policy reform. Decreasing seclusion has been found to significantly reduce aggression and time spent in medium security, while also increasing treatment engagement.^36^ FMHS patients have highlighted that prioritising patient contact and positive staff–patient relationships is the most effective strategy for reducing seclusion use.^36^ As reported by participants, external mechanisms, such as positive staff–patient interactions and access to more helpful coping mechanisms arising from greater freedoms (e.g. community leave), contributed to their recovery. Despite this, staff typically attribute harmful behaviours to internal patient factors (e.g. mental health diagnosis), rather than relational or organisational factors.^43,44^ It should be noted that restrictive practices are often necessary for patient and staff safety because of the risk of harm that may be posed by the individual to themselves or others.^16^ Reflective practice attended by staff from all levels of seniority, alongside constant supervision, can ensure restrictive practices are used appropriately.^45^ This could further promote a recovery-oriented, therapeutic-focused approach that allows patients to appropriately access healthy coping mechanisms that decrease risk of harmful behaviours.^45^ For example, meaningful activities, such as projects with other patients and staff members, physical activities, nature connection (e.g. gardening) and music production, are often available in FMHSs and have been shown to help patient recovery and interpersonal relationships.^46^ Moreover, joint decision-making processes that involve FMHS patients in their risk assessment and management could ensure fair, transparent procedures that increase patients’ understanding of clinical decisions.^45^ Doing so has been found to not only enhance risk management effectiveness, but also patient autonomy and agency.^37,41,47^ Such outcomes can reduce the risk of dual harm by addressing feelings of frustration, hopelessness and institutionalisation. Nevertheless, it should be noted that factors outside of staff's control, such as policies (e.g. risk-aversive security policies), hospital structure, overcrowding, staff shortages and lack of resources, may increase restrictiveness within the hospital and a sense of institutionalisation. As such, it is crucial for future work to consider how such issues can be addressed on an institutional and systemic level.

The present study emphasises the importance of individualised approaches that consider the multi-functionality and duality of patients’ self-harm and aggression.^16,48^ Participants highlighted that they engaged in self-harm and aggression for various shared purposes, including managing emotions and communicating distress. However, these behaviours also served diverging functions (e.g. self-harm as punishment, aggression as an act of defence). As such, it is important for staff within forensic settings to consider the shared and distinct functions that an individual's self-harm and aggression may serve, as well as how the presence of one behaviour may influence the risk of the other. Moreover, despite their range of mental health diagnoses, participants expressed common shared risk factors for their self-harm and aggression, including emotional dysregulation and self-worthlessness. Therefore, risk assessment tools could aim to identify such shared risk factors in those with a history of dual harm and sole harm, to assess the likelihood of self-harm and aggression co-occurring. Additionally, as opposed to common diagnosis-specific interventions, transdiagnostic approaches targeting the shared underlying processes and functionality of self-harm and aggression may be effective for those who engage in dual harm.^48^ For example, given that patients largely reported using self-harm and aggression interchangeably to regulate negative emotions, interventions targeting emotional dysregulation, such as dialectical behavioural therapy, may be helpful in addressing dual harm.

It is important to note the limitations of this study. There is a lack of consensus within the literature regarding the definition of dual harm.^47^ Therefore, the present study adopted a broad definition of this phenomenon that provided no specifications regarding the timing of the self-harm and aggressive acts. Future studies should focus on establishing a definition that will allow a standardised assessment of dual harm to be developed. Moreover, eligibility was based on a previous study. Although all participants expressed a cessation or reduction in their dual harm, this may have been an artefact of the eligibility criteria. Those who were too high of a risk to themselves or others to participate, and were therefore more likely to be engaging in dual harm, were unable to take part. Additionally, most participants were diagnosed with psychosis. Previous research has highlighted a link between mental health difficulties and dual harm.^9^ However, the role of mental health was not sufficiently brought up by participants to warrant a shared theme across the data. It may be that this link is more apparent among those who experience a greater severity of symptoms and who may therefore have been unable to participate in this study because they were unwell. Future research should investigate experiences of dual harm among those with distinct characteristics and patterns of harmful behaviours.

## Supporting information

Shafti et al. supplementary material Shafti et al. supplementary material

## Data Availability

Because of the qualitative nature of this research and in accordance with confidentiality procedures, data availability is not applicable to this article.
