# Supplementary material for: ‘If one doesn't happen, the other will’: forensic mental health service patients’ experiences of co-occurring self-harm and aggression
Source: BJPsych Open. 2025 Jan 22;11(1):e20. doi: 10.1192/bjo.2024.834 (PMC11822948; doi:10.1192/bjo.2024.834)
Supplement: Shafti et al. supplementary material [file S2056472424008342sup001.docx]

**Topic Guide:**

**Exploring how personality and emotion influence forensic mental health service users’ dual-harm thoughts/behaviours**

Introduction

- Thank you for agreeing to do this interview. The aim of this interview will be to find out about your experiences and how things like personality and emotion might explain some people’s difficulties with self-harm or aggressive behaviour. The interview should last about an hour. You’ll be able to take breaks if needed, and you won’t have to answer any questions you don’t want to.
- Clarify confidentiality
- Do you have any questions before we start?

Warm-up

1. Where are you from?
2. What are your hobbies/interests?
3. Why did you decide to take part in this study?

- *Prompt: Interested in the research topic? Wanted to do something different?*

1. What do you understand this study to be about?

Experience of emotions

1. First, I would like to ask you about your emotions. We can all struggle with strong feelings or emotions at certain times. Can you tell me about a time when you felt a particularly strong or troubling emotion, such as anger or sadness? Recent memories might be easier to talk about rather than going too far back into your history.

- *Prompt: are there any emotions you find particularly difficult? Have you felt angry, sad, frustrated, ashamed…lately? What sorts of things were happening for you around that time?*

1. When people experience strong emotions, they cope with these in different ways. Can you tell me what kind of things you have done to cope with the strong emotions you experienced?

- *Prompt: How did you make yourself feel better or less worse? How did you end any pain you might have been experiencing? Are there any ways you have tried to cope with difficult feelings that might have been more harmful to you?*

1. How has the way you deal with your emotions changed over time?
2. *[If there’s been a change]* What has caused this change?

- *Prompt: any key learning events?*

1. When you experienced those strong emotions, how much choice or control did you have over yourself or your actions?
   1. What helps or hinders how ‘in control’ you feel?
2. Everyone’s emotions are different. Some people’s feelings are stronger or weaker, some people’s feelings change more or less. How do you think your experience of emotions compares to others’?

- *Prompt: same or different? If different, how so? Types of feelings (anxiety, anger, frustration, revenge, low mood), frequency, instability, intensity?*

Self-harm

1. Last time we met, you mentioned that you have self-harmed before. Can you tell me about the most recent or memorable time you thought about or engaged in self-harm?
2. When was it? What sorts of things were happening around you at that time?
3. Can you remember how your self-harming started? What was on your mind at that time? Were you thinking about anything in particular?
4. What kinds of emotions or feelings were you experiencing before you self-harmed? Can you describe these to me?

- *Prompt: any negative or difficult feelings?*

1. Some people find it easier to draw how they felt on the inside, would you like to have a go at drawing your emotions before you self-harmed?
2. How did these feelings influence your self-harm (inflate / deflate your urge to act)? Did anything make you want to hurt yourself more or less?
3. How did self-harming affect your emotions or feelings? How was this different or similar to other times you self-harmed?

- *Prompt: did it help you in any way? Did it make things worse?*

1. How has your experience of self-harm changed over time? Do you use self-harm now in the same way that you used to do? How has this changed for you? In what ways?

Aggression

1. You’ve mentioned that you have been aggressive before. Can you tell me a little about a recent or memorable time you thought about or became aggressive?
2. When was it? What sort of things were happening around you at that time?
3. Can you remember how your aggressive behaviour started? What was on your mind? Were you thinking about anything in particular? Were you imagining anything?
4. What emotions were you experiencing before you were aggressive? Can you describe these to me?
5. Some people find it easier to draw how they felt on the inside, would you like to have a go at drawing your emotions before you self-harmed?
6. How did these feelings affect your behaviour? Did your feelings make you want to do something? What did you then do?

- *Prompt: How did these feelings influence you becoming aggressive (inflate / deflate your urge to act)?*

1. People can feel many different ways after they’ve been aggressive. How did you feel after you were aggressive?

- *Prompt: Negative or positive feelings?*

1. To what extent did these feelings you experienced differ from other times you were aggressive? What sorts of things influence how you feel after you’ve been aggressive?

- *Prompt: Consequences of behaviour? Who the victim is?*

1. How has your aggressive behaviour changed over time? In what ways?

Dual-harm

1. We’ve talked about how you have engaged in both self-harm and aggressive behaviour before. How different or similar are the situations that lead to these behaviours?

- *Prompt:* What sorts of situations lead to each of these behaviours?

1. To what extent do you feel you make a choice to either self-harm or be aggressive? How much control do you have over this decision to self-harm or become aggressive?

- *Prompt: How does each behaviour usually happen?*

1. How do you perceive your self-harm and aggression to be linked, if at all?
2. How do people tend to respond to your self-harm and aggression?

- *Prompt: counselling? Restraint? Isolation?*

a. How does this response affect your feelings or behaviour?

- *Prompt: Negative or positive feelings?*

1. How does being in mental health services affect your self-harming or aggressive thoughts and behaviours?
2. What, if anything, could have been done or should be done in the service to help you?

Personality

1. Now I’d like us to talk a little about your personality and what things were like for you growing up.

a. How would you say your personality is different to other people you know? What do you feel is distinct or different about you?

1. I’m interested to learn more about how you learned about your feelings and how to deal with them. What did your upbringing teach you about feelings and how to deal with them?
2. Growing up, what were your thoughts or attitudes about how people should deal with their emotions?
3. How have your beliefs about how you should deal with your emotions changed over time?
4. Growing up, what were your thoughts or attitudes about self-harming?
5. Did you witness self-harm around you?
6. How have your thoughts about self-harming changed over time?
7. Which thoughts do you think are more likely to contribute to your own self-harm behaviour?
8. Growing up, what were your thoughts or attitudes about aggressive behaviour?
9. Did you witness aggression around you?
10. How have your thoughts about aggression changed over time?
11. Which thoughts do you think are more likely to contribute to your own aggressive behaviour?
12. Is there anything else that you would like to say that I may have missed in the interview?

Wind-down questions (2-3 to be asked)

- Thank you for answering all these difficult questions. Can we now move onto some more every day questions?

1. Is there anything that helps you when you’re feeling emotional?
2. Have you found a good place to show your emotions? What do you do there?

*Prompt: e.g. gym, chaplaincy?*

1. How have you found it answering these questions today?
2. I know that this session might have been upsetting for you. Is there anything that you can do for the rest of the day to make you feel a little better?

*Prompt: e.g. read a book, television?*

1. Do you have any hobbies?

*Prompt: e.g. interests inside or outside of services? Sports? Tv programmes?*
